# The effectiveness and cost effectiveness of a hospital avoidance program in a residential aged care facility: a prospective cohort study and modelled decision analysis

**DOI:** 10.1186/s12877-020-01904-1

**Published:** 2020-12-07

**Authors:** Hannah E. Carter, Xing J. Lee, Trudy Dwyer, Barbara O’Neill, Dee Jeffrey, Christopher M Doran, Lynne Parkinson, Sonya R Osborne, Kerry Reid-Searl, Nicholas Graves

**Affiliations:** 1 Australian Centre for Health Services Innovation and Centre for Healthcare Transformation, Queensland University of Technology, Brisbane, Australia; 2 Central Queensland University, Rockhampton, Australia; 3 University of Connecticut, Storrs, USA; 4 PresCare, Brisbane, Australia; 5 Cluster for Resilience and Wellbeing, Central Queensland University, Brisbane, Australia; 6 School of Medicine and Public Health, University of Newcastle, Newcastle, Australia; 7 School of Nursing and Midwifery, Centre for Health Research, Institute for Resilient Regions, University of Southern Queensland, Ipswich, Australia; 8 Duke NUS-Medical School, Singapore, Singapore

## Abstract

**Background:**

Residential aged care facility residents experience high rates of hospital admissions which are stressful, costly and often preventable. The EDDIE program is a hospital avoidance initiative designed to enable nursing and care staff to detect, refer and quickly respond to early signals of a deteriorating resident. The program was implemented in a 96-bed residential aged care facility in regional Australia.

**Methods:**

A prospective pre-post cohort study design was used to collect data on costs of program delivery, hospital admission rates and length of stay for the 12 months prior to, and following, the intervention. A Markov decision model was developed to synthesize study data with published literature in order to estimate the cost-effectiveness of the program. Quality adjusted life years (QALYs) were adopted as the measure of effectiveness.

**Results:**

The EDDIE program was associated with a 19% reduction in annual hospital admissions and a 31% reduction in the average length of stay. The cost-effectiveness analysis found the program to be both more effective and less costly than usual care, with 0.06 QALYs gained and $249,000 health system costs saved in a modelled cohort of 96 residents. A probabilistic sensitivity analysis estimated that there was an 86% probability that the program was cost-effective after taking the uncertainty of the model inputs into account.

**Conclusions:**

This study provides promising evidence for the effectiveness and cost-effectiveness of a nurse led, early intervention program in preventing unnecessary hospital admissions within a residential aged care facility. Further research in multi-site randomised studies is needed to confirm the generalisability of these results.

**Supplementary Information:**

The online version contains supplementary material available at 10.1186/s12877-020-01904-1.

## Background

As individuals live longer and healthier lives, there is growing demand for aged care services internationally [1–4]. In Australia, admissions to aged care services have increased by 31% over the last decade [5]. It is known that residents of residential aged care facilities (RACF) are frequent users of hospital services, with annual rates of more than 30 hospital transfers per 100 RACF beds commonly reported [6]. These admissions have been estimated to account for 3% of all hospital bed days [7].

Hospital admissions in this cohort are considered stressful, costly and are often unnecessary or potentially preventable [8–14]. Residents and their families express a preference for care to be provided in their home [15], and older people treated in these settings are less likely to experience complications commonly incurred during hospitalisation [6, 16–18]. Previous studies have found that RACF nursing staff have a genuine desire to care for their acutely unwell residents within the facility [19–22]. There is therefore a strong clinical and economic basis for hospital avoidance interventions that promote appropriate nursing care within the RACF.

A range of approaches to hospital avoidance in the RACF setting have been documented, including interventions to strengthen the interface between the RACF and the emergency department, hospital based out-reach programs, advance care planning initiatives and vaccination programs [6, 23]. Evidence is emerging that hospital admissions from the RACF can be reduced by implementing models of care that improve nursing staff confidence, clinical skills and access to resources [6, 23–28]. Previous studies have focussed on the impact of these programs on emergency department (ED) transfers and hospital admissions, with few reporting on changes to average length of stay for admitted patients. There is no published evidence on the cost-effectiveness of making these changes to models of care.

The Early Detection of Deterioration In Elderly residents (EDDIE) program is a hospital avoidance intervention aimed at improving the proactive care and management of residents by RACF nursing staff. It was originally developed by nursing staff within a regional Queensland RACF with input from community health care providers, with a retrospective evaluation demonstrating it was effective in reducing hospital admissions and had support among staff [29]. The program was then introduced in a second regional Queensland RACF within the framework of an implementation and cost-effectiveness research study. This paper reports on a prospective evaluation to (1) estimate the impact of the EDDIE program on hospital admission rates and length of stay; and (2) estimate the cost-effectiveness of the EDDIE intervention as compared to usual care.

## Methods

A prospective pre-post cohort study design was adopted to estimate the changes to hospital admission rates and length of stay in the 12 months pre and post-implementation of the EDDIE intervention in a 96 bed regional Australian RACF in June 2016. Participants included all residents within the facility over the study period. This represented a range of 91 to 96 residents, with an average monthly occupancy of 94 residents observed across both the pre and post EDDIE cohorts. We refer to residents present during the 12 months post implementation of the EDDIE intervention as the intervention cohort (June 2016 – May 2017), and residents present during the 12 months prior to the EDDIE interventions as the usual care cohort (June 2015–May 2016). We used the CHEERS checklist as our reporting guide [30].

Individual patient demographic data were not collected from the RACF as part of this study. To inform the generalisability of our results we obtained key descriptive statistics about the population of aged care residents within the immediate geographic region from an administrative database [31]. These data indicate that across the 9 RACFs operating within the immediate geographic region in 2017, 65% of residents were female and over 50% were aged 85 and above. The average length of stay for patients who died in the facility was 37.8 months, and 48.5% of residents had a diagnosis of dementia. A detailed summary of the population characteristics is included in Additional File 1.

### The intervention

The EDDIE program was developed to enable practice change and improvement so that deteriorating residents could be identified early and managed proactively within the RACF, reducing the need for transfer to hospital, or shortening length of stay. Importantly, the intervention did not involve the employment of additional nursing staff within the RACF. The focus was instead on upskilling existing staff members and empowering them to manage sub-acute episodes within the facility. In the context of this study, a sub-acute episode was defined as a scenario where a resident required more intensive treatments, interventions and/or frequent assessments for a complex condition that did not require immediate hospitalisation. This included conditions such as kidney infections, pneumonia and urinary tract infections where residents could be monitored using appropriate diagnostic equipment and treated with intra-venous antibiotics within the RACF. While existing nursing staff were qualified to manage sub-acute episodes within the facility, it had not been common practice until the implementation of the EDDIE program.

The intervention encompassed four core components:

1. ***Advanced clinical skills training for all nursing and care staff:*** Training was mandatory and involved an initial face-to-face education session on the early identification of deterioration, and appropriate clinical response. Targeted training was also provided on clinical management of the eight conditions that had been identified as likely to result in avoidable hospitalisation: urinary tract infections, chest pain, falls, delirium, dehydration, dyspnoea, constipation, palliative care.
2. ***Decision support:*** A decision support tool in the form of a flip chart was readily available to staff within the RACF, as well as in pocket-size books that staff could carry on their person. This tool reinforced the educational content and was structed around a ‘traffic light’ system where colour-coded parameters were established on assessment documentation to determine a change in health status, which then triggered further assessment and treatment. The traffic light tool included specific clinical decision making guidelines for managing acute deterioration across all eight conditions identified and addressed within the training (listed under point (1) above). A track and trigger tool was used to monitor vital signs. A standard communication approach (‘Situation, Background, Assessment, Recommendation’) was used for written and oral communication [32].
3. **Diagnostic medical equipment:** Diagnostic equipment, not commonly found in the RACF setting, was introduced at the study site to support nursing staff to monitor residents at the early stages of deterioration. This included bladder scanners, ECG machines, vital signs monitors and pulse oximeters. Use of the equipment was covered in the mandatory face to face training sessions, with ongoing, on-the-job training opportunities with a nurse educator also available to staff.
4. **Specialist clinical support and collaboration**, grounded in the principles of implementation science through the adoption of the i-PARiHS implementation framework [33]. This included a knowledgeable and enthusiastic on-site clinical leader; a number of clinical ‘champions’ to promote staff uptake and adoption; and, targeted engagement with external stakeholders including: General Practitioners and their practice nurses; nurse practitioners; hospital staff including geriatricians and emergency department staff; ambulance staff and residents’ families. Embedding of the program into business-as-usual practices was achieved through the development of clinical policies and procedures within the RACF to support the use of clinical decision support tools and program pathways.

### Statistical analysis of the observed hospital admissions data

There was one hospital admission that occurred within the usual care period but where discharge occurred after EDDIE implementation. This admission was analysed as part of the usual care cohort in keeping with the EDDIE program’s focus on hospital avoidance. The impact on variation in the data was explored by fitting statistical distributions around key results based on the observed means and standard deviations from both intervention and usual care cohorts. A normal distribution provided the best fit for the number of admissions per annum. A gamma distribution was used to represent length of stay as its positive, right-skewed nature accounted for a small proportion of admissions experiencing relatively long lengths of stay.

### Costs of implementation

A set of the initial implementation costs of EDDIE were estimated based on the project data collection. The decision support tool was developed and piloted in a previous study and the costs associated with this were not included in this analysis. We accounted for the cost of printing the decision support materials, as well as the staff costs associated with the implementation strategy such as training, stakeholder engagement and project management activities. Details of the time spent on these activities, as well as the numbers of type of staff members involved, were prospectively collected in an implementation activity log. The costs of staff time were assigned using published salary band data where available. These costs are reported in Additional File 2. Due to the one-off, upfront nature of these costs they were not included in the modelled analysis.

### Modelled cost-effectiveness analysis

A Markov model was developed to estimate the cost-effectiveness of the EDDIE intervention compared to usual care over a period of 12 months. The model defined a number of discrete health states that aged care residents could experience over a period of 365 days including: time spent within the RACF as a stable resident; ‘sub-acute episodes’ involving management of resident deterioration within the RACF; hospital admissions; and death. A set of transition probabilities governed the likelihood of residents transitioning from one state to another at the end of each daily cycle. The Markov model structure is included in Additional File 3.

The model was used to synthesise data collected in the study with published literature on the outcomes associated with relevant health states experienced by residents. Cost-effectiveness was assessed by comparing the incremental differences in costs and quality adjusted life years (QALYs) for the intervention cohort relative to the usual care cohort. QALYs were derived by weighting the time spent in each health state by a health related utility associated with that state. Utilities are values that represent the strength of individuals’ preferences for different health states. They are anchored by a scale where a utility of zero is equivalent to death and a utility of 1 is equivalent to full health [34]. A period of 10 years spent in a health state with a utility of 0.6 would therefore represent 6 QALYs. The evaluation was conducted from the perspective of the Australian health care system in which aged care services and hospital admissions are publicly funded. All costs are reported in 2018 Australian dollars.

All probabilities, costs and utility values applied in the model, along with respective standard deviations and data sources where relevant, are reported in Table 1. Data collected prospectively throughout the study was used to populate probabilities of transitioning between the different health states in the model, and to assign the costs of equipment. RACF bed day costs, hospital costs and utility values were estimated from the published literature.

**Table 1:** Transition probabilities applied in the cost-effectiveness model

Parameters	Base case estimate	SD	Source
**Transition probabilities:**			
Intervention cohort			
Daily probability of sub-acute episode	0.003	0.007	Study data
Proportion of sub-acute episodes treated within the facility	0.670	0.388	Study data
Daily probability of sub-acute episodes admitted to hospital	0.722	0.288	Study data
Daily probability of residents being discharged from hospital	0.283	0.150	Study data
Usual care cohort			
Daily probability of residents being admitted to hospital	0.001	0.004	Study data
Daily probability of residents being discharged from hospital	0.151	0.072	Study data
All residents			
Daily probability of death	0.0011	0.0001	Study data
**Costs**			
New diagnostic equipment (annualised)^a^			
Bladder Scanner ×1	1714	672	Study data
ECG Machine ×1	351	138	Study data
Vital Signs Monitor ×1	277	109	Study data
RACF bed day	194	76	[35]
Ambulance transfer to hospital	649	254	[36]
Hospital bed day	1807^b^	1028	[14]
**Utility values**			
RACF residents	0.514	0.252	[37]
Elderly inpatients admitted from RACF	0.44	0.4	[38]

*RACF* residential aged care facility; *SD* standard deviation; *ECG* electrocardiogram

^a^Costs were annualised over a useful life of 7 years according to Australian government depreciation schedules (Income Tax Assessment Act, Income Tax (Effective Life of Depreciating Assets) Determination 2015)

^b^Inflated to 2018 dollars using an index of hospital price inflation [39]

Transition probabilities were derived from the observed daily events data collected at the RACF over the period June 2015–May 2016 for usual care and June 2016 – May 2017 for the EDDIE intervention.

Costing items included the cost of additional diagnostic equipment not typically utilised in the RACF setting that were purchased in order for trained staff to better detect and manage sub-acute episodes. Equipment costs were annualised over a period of 7 years, reflecting their useful life as defined in the Australian government depreciation schedules [40]. A cost per day was assigned to RACF bed days based on current national fee schedules [35]. The cost of a hospital bed day was informed by a 2011 Australian study that produced estimates of admissions costs and length of stay that were specific to a RACF cohort [14]; this was then inflated to 2018 dollars using an index of hospital price inflation [39]. The cost of an ambulance transfer was also assigned with each hospital admission in line with standard practice [36].

The model assigned separate utility values according to whether a resident was in the RACF or in hospital.

### Sensitivity analysis

A probabilistic sensitivity analysis was performed in order to estimate the impact of simultaneous uncertainty across all modelled estimates. A normal distribution was applied to cost parameters with a 95% confidence interval encompassing a variation of 20% above and below the base case estimate. The exception was the cost per hospital bed day which was assigned a gamma distribution (SD 1028) based on the nature and availability of these data [14]. Beta distributions were fitted to the transition probability and utility estimates using the standard deviations reported in Table 1. A Monte Carlo simulation was then performed with 1000 randomly drawn samples taken from each of the modelled parameter distributions.

The modelled uncertainty was represented in the form of a distribution around the Net Monetary Benefit (NMB) associated with a decision to adopt the EDDIE intervention. This provides a measure of the value of the intervention in monetary terms when the willingness to pay for a QALY is known. A positive NMB indicates that an intervention is cost-effective. The NMB was estimated using a recently published study of the optimal willingness to pay for a QALY in an Australian setting of $28,000 [41]. A sensitivity analysis estimated the cost-effectiveness of the intervention where the willingness to pay for health benefits was set to zero.

## Results

There were 112 sub-acute episodes recorded in the intervention cohort over 12 months, with 75 of these treated within the RACF only. The remaining 37 sub-acute events resulted in hospital admissions with a mean length of stay of 4.8 days. In comparison, a total of 45 hospital admissions over 12 months were recorded in the usual care cohort with a mean length of stay of 7.7 days. This represented a 19% reduction in annual hospital admissions and a 31% reduction in the average length of stay following implementation of the EDDIE intervention. Additional File 4 presents the probability density functions around both the admission rates and length of stay outcomes.

The modelled cost-effectiveness analysis estimated that the EDDIE intervention was dominant relative to usual care; that is, it was associated with additional QALYs and reduced costs. When extrapolated to a 96 bed RACF, assuming full bed capacity over a 12 month period, the intervention would prevent 9 hospital admissions and result in 154 fewer bed days (Table 2). This translated to a total cost saving of $249,000. The incremental QALYs gained was positive, but modest at 0.06 QALYs per 96 residents. This was due to the relatively small decrement in utility associated with hospital admissions when compared to the baseline utility score of RACF residents.

**Table 2:** Mean cost-effectiveness outcomes taken from 1000 Monte Carlo simulations modelled over 12 months in a cohort of 96 residents

Modelled outcomes per 96 residents	Intervention	Usual care	Difference
Number of admissions	26	35	−9
Total hospital bed days	132	286	−154
Total costs ($000’s)	5941	6190	−249
Total QALYs	39.75	39.69	0.06
Cost-effectiveness result	Intervention is dominant^a^		

^a^A cost-effectiveness result of ‘dominant’ indicates an intervention is both more effective and less costly than the alternative

**Fig. 1:**
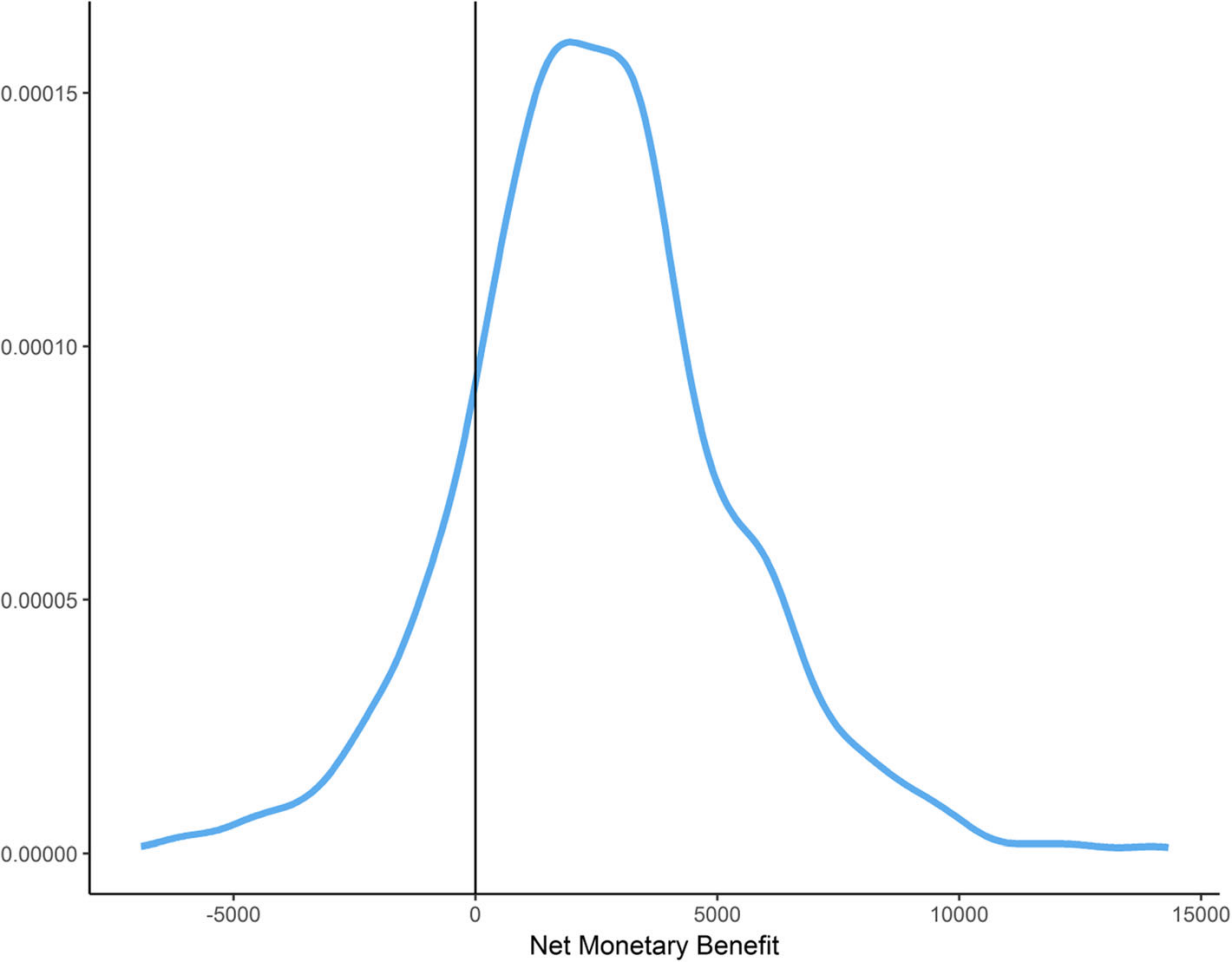
Distribution of Net Monetary Benefit across 1000 Monte Carlo Simulations. The Net Monetary Benefit calculation is based on a willingness to pay of $28,000 per quality adjusted life year (QALY)

The mean NMB of the EDDIE intervention over 1000 Monte Carlo simulations was $2611 per resident (SD $2802) when adopting a willingness to pay of $28,000 per QALY [41]. Figure 1 presents the distribution of NMB samples. Approximately 86% of the simulations produced a positive NMB, providing a high likelihood that the decision to adopt the EDDIE intervention was cost-effective. When an alternate willingness to pay of $0 per QALY was adopted, a mean NMB of $2506 (SD $2799) was estimated with 85% of simulations remaining positive.

## Discussion

The 12 months following the commencement of the EDDIE intervention was associated with a 19% reduction in annual hospital admissions and a 31% reduction in the average length of stay per admission when compared to the previous 12 months. When outcomes were modelled in a cohort of 96 RACF residents the intervention produced an additional 0.06 QALYs while saving $249,000 to the health care system. After accounting for plausible uncertainty in the model, there was an 86% chance of the intervention being cost-effective when adopting a willingness to pay of $28,000 per QALY. When the willingness to pay for health benefits was assumed to be zero, there was still an 85% change of the intervention being cost-effective and in this case, cost-saving to the health care system.

In September 2018, the Australian Government announced a Royal Commission into Aged Care Quality and Safety, in recognition of the need for higher quality residential and in-home aged care that meets community expectations. An interim report from the Commission entitled ‘Neglect’ has identified multiple systemic failures, including a “workforce that is under pressure and under-appreciated and that lacks key skills” [42]. Among other issues, the interim report noted that the quality of care received by people in aged care is highly dependent on the quality of the paid carers, their working conditions, their leadership and engagement. While it is clear that the aged care system in Australia requires fundamental reform, this will need to be guided by evidence-based approaches. The findings presented here contribute to this emerging evidence base.

This is the first economic evaluation of a hospital avoidance intervention in the aged care setting. Prospective data were collected on the number of subacute episodes managed within the RACF as well as on the implementation costs of the intervention, including staff time spent on training, stakeholder engagement and project management activities. This information may be valuable to other RACFs considering adopting a similar program.

A notable finding was that the EDDIE program was associated with a shorter length of stay for residents who were admitted. This is despite the reasonable assumption that the residents who were admitted to hospital may be higher acuity or in need of more specialist care. Shorter lengths of stay may be explained in part due to increased hospital staff confidence in the ability of the RACF to provide clinical care for patients with complex health needs. As part of the EDDIE program, the RACF engaged with nearby hospitals and educated hospital staff about the higher level of care and diagnostic equipment available. In one case, a hospitalized resident described as “complex” who required frequent bladder scans was returned to the RACF because the hospital discharge staff knew that the equipment and expertise to manage the patient’s care were available.

The study was limited to a single RACF in a regional area, and it is therefore unknown how the results we have reported may translate to other settings. A further limitation was that it was unethical and impractical to randomise intervention provision as it is added to the current model of care provision. As the intervention and usual care cohorts encompassed non-static resident populations it was not feasible to summarise and control for resident characteristics across the pre and post intervention periods. The analysis would have been further strengthened by the collection of prospective utility data which may be more sensitive to changes in the overall quality of care provided within the RACF.

Our findings support the growing body of evidence to suggest that programs allowing for sub-acute care to be provided within the RACF setting may improve health service outcomes. Previous Australian studies have evaluated hospital in the nursing home programs or other hospital or emergency department (ED) led outreach services that assist with the assessment of deteriorating residents [26, 43, 44]. These evaluations have reported significant reductions in ED transfers and hospital admission rates, but did not assess cost-effectiveness. The EDDIE intervention was instead focussed on upskilling existing RACF nursing staff and empowering them to proactively detect and respond to early signs of resident deterioration. In this sense it takes a similar approach to the hospital avoidance program ‘Interventions to Reduce Acute Care Transfers’ (INTERACT II) developed in the United States. This program was initially reported to have reduced hospital admissions by 17–24% across 24 nursing homes [24]. However, a more recent cluster randomised trial in 84 nursing homes found that the INTERACT program did not have a statistically significant effect on hospital admissions or ED transfer rates [45]. The authors suggested several factors that may have contributed to the difference in findings between the studies, including: the randomisation process eliminating a potential self-selection bias in the earlier study; a change in the policy climate which meant that some aspects of the INTERACT program were potentially adopted in the control groups; and less intensive training and support available in the larger trial. A qualitative evaluation found that commonly cited barriers to implementation being scarce resources, staff resistance, competing demands and instability of nursing home leadership [45].

The mixed nature of findings from previous hospital avoidance studies highlight the challenges associated with implementation of quality improvement initiatives in the aged care setting. It will be important for future studies to consider context-specific factors that may impact on the scale-up and sustainability of such programs, recognising the scope for an intervention such as EDDIE to be tailored according to the needs of individual sites. Harvey et al. have highlighted the need for process evaluations within health service implementation studies to balance between ‘fidelity’ to the core intervention with pragmatic considerations around the adaptation of interventions [46]. The authors recommended that fidelity be more broadly framed around mechanisms of change, informed by prospective use of process evaluation data and thorough investigation of the context-facilitation dynamic. This will enable insights into not only whether an intervention is effective, but why it may be effective in some settings and not others. Future studies of hospital avoidance programs in this setting should also focus on collecting self- or proxy-reported resident outcomes data, including on quality of life, where there remains a lack of evidence.

## Conclusions

The results of this evaluation are encouraging and provide compelling evidence to support the effectiveness of the EDDIE program in facilitating the delivery of sub-acute care by nursing staff in the RACF setting. The provision of a simple decision aid and staff training is a low cost intervention that may improve the quality of care residents receive while simultaneously providing high value to health systems by reducing the morbidity and expense associated with hospital transfers and admissions. Further implementation and evaluation of the EDDIE program in multi-site, randomised controlled studies is warranted to build a stronger evidence base around its effectiveness and cost-effectiveness. Rigorous process evaluation should accompany future multi-site studies to determine context-specific barriers and facilitators to implementation, and how these may be addressed.

## Supplementary Information

**Additional file 1. **Characteristics of the RACF population in the immediate geographic region.

**Additional file 2. **Costs of EDDIE implementation.

**Additional file 3. **Cost-effectiveness model structure. *Figure A:* Health states and transitions within usual care. *Figure B:* Health states and transitions within the EDDIE intervention.

**Additional file 4. **Figure A: Density of the fitted Normal distribution for the annual number of hospital admissions. Figure B: Density of the fitted Gamma distribution for length of stay.

## Data Availability

The full, de-identified, dataset used in the analysis is available from the corresponding author upon request.

## Abbreviations

RACF: Residential Aged Care Facility
ED: Emergency Department
EDDIE: Early Detection of Deterioration In Elderly residents
RADD: Residential Acute Deterioration Detection
QALY: Quality Adjusted Life Year
NMB: Net Monetary Benefit
SD: Standard Deviation

## References

[1] Palangkaraya A, Yong J (2009). Population ageing and its implications on aggregate health care demand: empirical evidence from 22 OECD countries. Int J Health Care Finance Econ.

[2] Koopmans R, Lavrijsen J, Hoek J, Went P, Schols J (2010). Dutch elderly care physician: a new generation of nursing home physician specialists. J Am Geriatr Soc.

[3] Gomes B, Higginson I (2008). Where people die (1974–2030): past trends, future projections and implications for care. Palliat Med.

[4] Rechel B, Doyle Y, Grundy E, McKee M (2009). How can health systems respond to population ageing? Technical report. In.

[5] Australian Government Productivity Commission: Report on Government Services 2018. In*.* Edited by Commonwealth of Australia. Canberra; 2018: Chapter 14: Aged Care Services.

[6] Arendts G, Howard K (2010). The interface between residential aged care and the emergency department: a systematic review. Age Ageing.

[7] Hillen JB, Reed RL, Woodman RJ, Law D, Hakendorf PH, Fleming BJ (2011). Hospital admissions from residential aged care facilities to a major public hospital in South Australia (1999–2005). Australas J Ageing.

[8] Spector WD, Limcangco R, Williams C, Rhodes W, Hurd D (2013). Potentially avoidable hospitalizations for elderly long-stay residents in nursing homes. Med Care.

[9] Ouslander JG, Lamb G, Perloe M, Givens JH, Kluge L, Rutland T, Atherly A, Saliba D (2010). Potentially avoidable hospitalizations of nursing home residents: frequency, causes, and costs. J Am Geriatr Soc.

[10] Grabowski DC, O'Malley AJ, Barhydt NR (2007). The costs and potential savings associated with nursing home hospitalizations. Health Aff (Millwood).

[11] Creditor MC (1993). Hazards of hospitalization of the elderly. Ann Intern Med.

[12] Dwyer R, Gabbe B, Stoelwinder JU, Lowthian J (2014). A systematic review of outcomes following emergency transfer to hospital for residents of aged care facilities. Age Ageing.

[13] Arendts G, Popescu A, Howting D, Quine S, Howard K (2013). ‘They never talked to me about…’: perspectives on aged care resident transfer to emergency departments. Australasian J Ageing.

[14] Close J, Lord S, Antonova E, Martin M, Lensberg B, Taylor M, Hallen J, Kelly A (2012). Older people presenting to the emergency department after a fall: a population with substantial recurrent healthcare use. Emerg Med J.

[15] Carusone S, Loeb M, Lohfeld L (2006). Pneumonia care and the nursing home: a qualitative descriptive study of resident and family member perspectives. BMC Geriatr.

[16] Boockvar K, Gruber-Baldini A, Burton L, Zimmerman S, May C, Magaziner J (2005). Outcomes of infection in nursing home residents with and without early hospital transfer. J Am Geriatr Soc.

[17] Caplan G, Ward J, Brennan N, Coconis J, Board N, Brown A (1999). Hospital in the home: a randomised controlled trial. Med J Aust.

[18] Cheng J, Montalto M, Leff B (2009). Hospital at home. Clin Geriatr Med.

[19] Stokoe A, Hullick C, Higgins I, Hewitt J, Armitage D, O’Dea I (2015). Caring for acutely unwell older residents in residential aged care facilities: perspectives of staff and general practitioners. Aust J Ageing.

[20] O'Neill BJ, Dwyer T, Reid-Searl K, Parkinson L (2017). Managing the deteriorating nursing home resident after the introduction of a hospital avoidance programme: a nursing perspective. Scand J Caring Sci.

[21] O'Neill B, Reid-Searl K, Dwyer T, Parkinson L (2017). The deteriorating resident in residential aged care: a focus group study. Collegian.

[22] O'Neill B, Parkinson L, Dwyer T, Reid-Searl K (2015). Nursing home nurses' perceptions of emergency transfers from nursing homes to hospital: a review of qualitative studies using systematic methods. Geriatr Nurs.

[23] Graverholt B, Forsetlund L, Jamtvedt G (2014). Reducing hospital admissions from nursing homes: a systematic review. BMC Health Serv Res.

[24] Ouslander JG, Lamb G, Tappen R, Herndon L, Diaz S, Roos BA, Grabowski DC, Bonner A (2011). Interventions to reduce hospitalizations from nursing homes: evaluation of the INTERACT II collaborative quality improvement project. J Am Geriatr Soc.

[25] Tena-Nelson R, Santos K, Weingast E, Amrhein S, Ouslander J, Boockvar K (2012). Reducing potentially preventable hospital transfers: results from a thirty nursing home collaborative. J Am Med Dir Assoc.

[26] Crilly J, Chaboyer W, Wallis M, Thalib L, Polit D (2011). An outcomes evaluation of an Australian Hospital in the Nursing Home admission avoidance programme. J Clin Nurs.

[27] Conway J, Higgins I, Hullick C, Hewitt J, Dilworth S (2015). Nurse-led ED support for residential aged care facility staff: an evaluation study. Int Emerg Nurs.

[28] Street M, Considine J, Livingston P, Ottmann G, Kent B (2015). In-reach nursing services improve older patient outcomes and access to emergency care. Australas J Ageing.

[29] Parkinson L, O’Neill B, Dwyer T, Reid-Searl K. Recognising and responding to the deteriorating aged care client: evaluation of the PresCare SubAcute care (PCSAC) project. Final report to PresCare. Central Queensland University. 2015.

[30] Husereau D, Drummond M, Petrou S, Carswell C, Moher D, Greenberg D, Augustovski F, Briggs AH, Mauskopf J, Loder E (2013). Consolidated Health economic evaluation reporting standards (CHEERS) statement. BMJ.

[31] Australian Institute of Health and Welfare (2019). Gen Aged Care Data. In.

[32] Haig KM, Sutton S, Whittington J (2006). SBAR: a shared mental model for improving communication between clinicians. Jt Comm J Qual Patient Saf.

[33] Harvey G, Kitson A: PARIHS revisited: from heuristic to integrated framework for the successful implementation of knowledge into practice. Implement Sci 2015, 11(1):1–13. 10.1186/s13012-016-0398-2 PMC4807546 27013464

[34] Torrance G (1986). Measurement of health state utilities for economic appraisal: a review. J Health Econ.

[35] Australian Government Department of Health (2018). Schedule of Fees and Charges for Residential and Home Care: From 20 March 2018. Canberra.

[36] Queensland Health (2017). The State of Queensland (Queensland Health) annual report 2016–17. Edited by Department of Health. Brisbane.

[37] Gordon A, Franklin M, Bradshaw L, Logan P, Elliott R, Gladman J (2014). Health status of UK care home residents: a cohort study. Age Ageing.

[38] Hickson M, Frost G (2004). An investigation into the relationships between quality of life, nutritional status and physical function. Clin Nutr.

[39] Australian Bureau of Statistics (2018). Consumer Price Index, Australia, March 2018. vol. Cat, no. 6401.1. Canberra.

[40] Australian Government (2015). Income Tax Assessment Act, Income Tax (Effective Life of Depreciating Assets) Determination.

[41] Edney L, Afzali H, Cheng T, Karnon J (2018). Estimating the reference incremental cost-effectiveness ratio for the Australian health system. PharmacoEconomics.

[42] Royal Commission into Aged Care Quality and Safety. Neglect. Interim Report. Canberra: Interim Report; 2019.

[43] Fan L, Hou XY, Zhao J, Sun J, Dingle K, Purtill R, Tapp S, Lukin B (2016). Hospital in the Nursing Home program reduces emergency department presentations and hospital admissions from residential aged care facilities in Queensland, Australia: a quasi-experimental study. BMC Health Serv Res.

[44] Hullick C, Conway J, Higgins I, Hewitt J, Dilworth S, Holliday E, Attia J (2016). Emergency department transfers and hospital admissions from residential aged care facilities: a controlled pre-post design study. BMC Geriatr.

[45] Kane RL, Huckfeldt P, Tappen R, Engstrom G, Rojido C, Newman D, Yang Z, Ouslander JG (2017). Effects of an intervention to reduce hospitalizations from nursing homes: a randomized implementation trial of the INTERACT program. JAMA Intern Med.

[46] Harvey G, McCormack B, Kitson A, Lynch E, Titchen A (2018). Designing and implementing two facilitation interventions within the ‘Facilitating Implementation of Research Evidence (FIRE)’study: a qualitative analysis from an external facilitators’ perspective. Implementation Science..

