# Supplementary material for: The effectiveness and cost effectiveness of a hospital avoidance program in a residential aged care facility: a prospective cohort study and modelled decision analysis
Source: BMC Geriatr. 2020 Dec 7;20:527. doi: 10.1186/s12877-020-01904-1 (PMC7720399; doi:10.1186/s12877-020-01904-1)
Supplement: Supplementary file 1 — Additional file 1. Characteristics of the RACF population in the immediate geographic region. [file 12877_2020_1904_MOESM1_ESM.pdf]

Characteristics of the RACF population in the immediate geographic region

RACF population characteristics	Regional average (N = 9 RACFs, 822 places)
**Male (%)**	**34.9**
100+	0.4
95–99	2.7
90–94	16.0
85–89	25.1
80–84	23.6
75–79	15.6
70–74	6.1
65–69	5.7
60–64	3.4
55–59	1.1
50–54	0.4
0–49	0.0
**Female (%)**	**65.1**
100+	1.2
95–99	8.8
90–94	21.2
85–89	27.1
80–84	18.6
75–79	9.4
70–74	7.6
65–69	2.2
60–64	1.8
55–59	1.2
50–54	0.6
0–49	0.2
**Average length of stay by discharge destination (months)**	
Death	37.8
Other facility	15.9
Hospital	0.5
Home/community	15.0
Other facility	0.5
Places in residential aged care per 1,000 people aged 70+	73.6
RACF residents with a dementia diagnosis (%)	48.5

RACF = residential aged care facility
