# Supplementary material for: The effectiveness and cost effectiveness of a hospital avoidance program in a residential aged care facility: a prospective cohort study and modelled decision analysis
Source: BMC Geriatr. 2020 Dec 7;20:527. doi: 10.1186/s12877-020-01904-1 (PMC7720399; doi:10.1186/s12877-020-01904-1)
Supplement: Supplementary file 2 — Additional file 2. Costs of EDDIE implementation. [file 12877_2020_1904_MOESM2_ESM.pdf]

Costs of EDDIE implementation

Implementation costs	$AUD
Decision support tool – printing costs	360
Project staff time on implementation activities	
Training and development (67 hrs)	*2793*
Stakeholder engagement (12 hrs)	*494*
Project management and leadership (14 hrs)	*648*
**Total**	**4,295**
