# Supplementary material for: The effectiveness and cost effectiveness of a hospital avoidance program in a residential aged care facility: a prospective cohort study and modelled decision analysis
Source: BMC Geriatr. 2020 Dec 7;20:527. doi: 10.1186/s12877-020-01904-1 (PMC7720399; doi:10.1186/s12877-020-01904-1)

Model Structure

A: Health states and transitions within usual care: *All residents begin the model in the residential aged care facility (RACF). Residents who deteriorate are admitted to hospital for treatment, and return to the RACF once stable. Residents may die in the RACF or in hospital.*

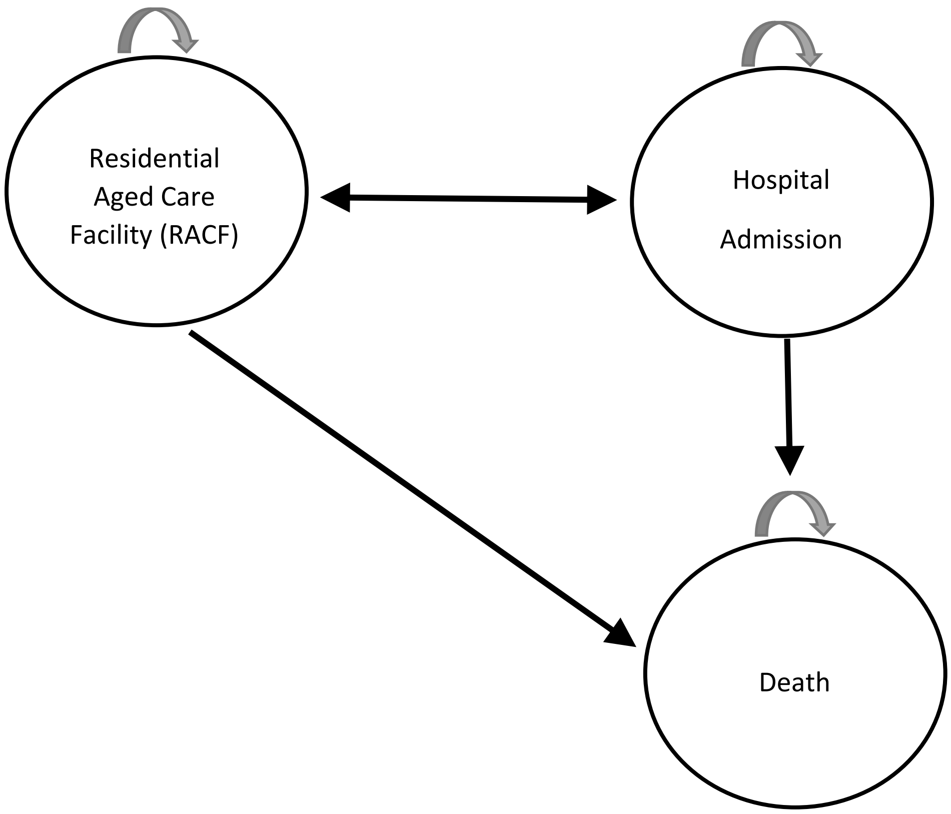

B: Health states and transitions within the EDDIE intervention: *All residents begin the model as stable in RACF. Residents who deteriorate are first managed as a sub-acute episode within the RACF, where they may then stabilise and remain within the RACF. Residents who deteriorate further are admitted to hospital for treatment, and return to the RACF once stable. Residents may die at any point in the model.*

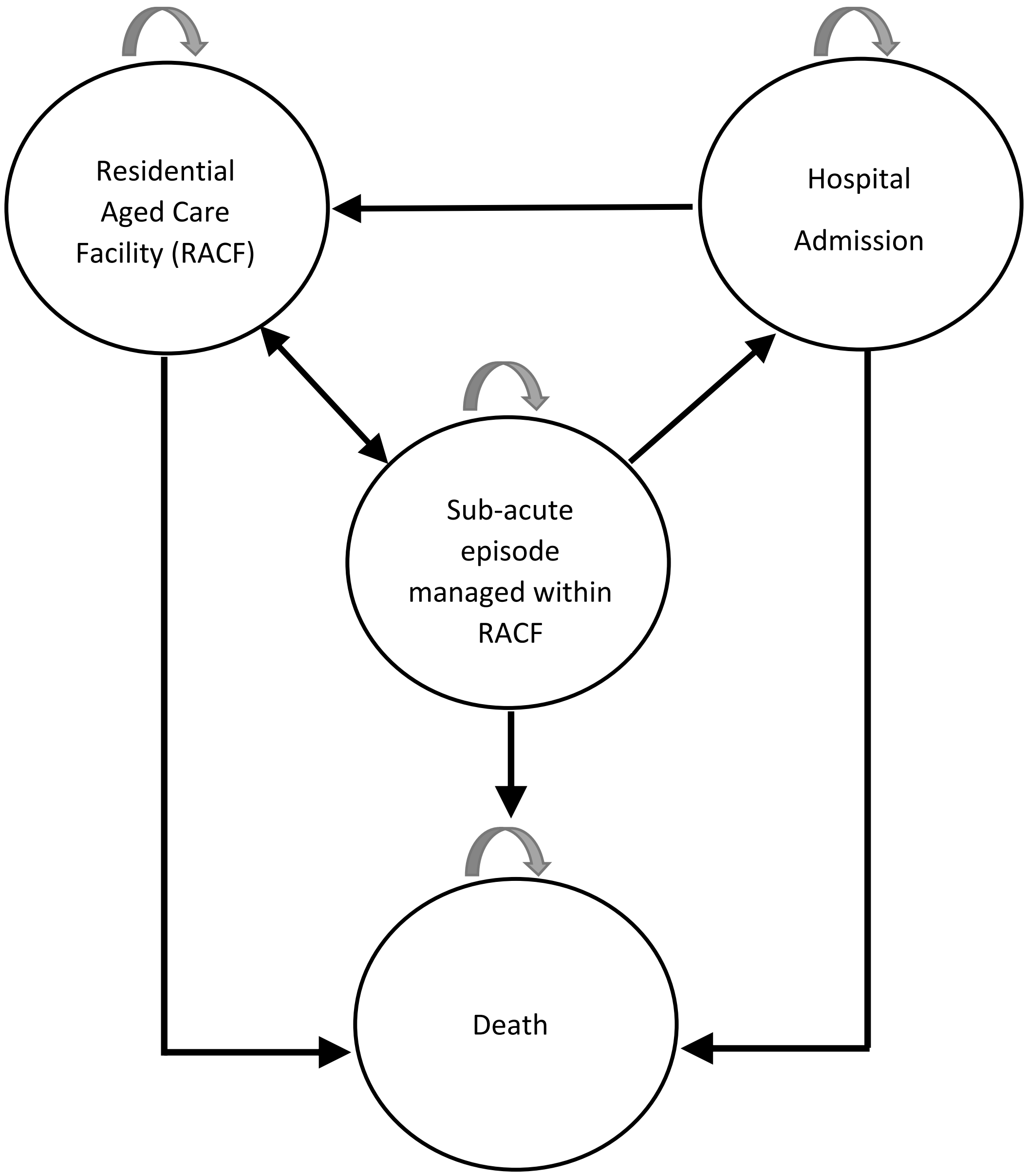

Supplement: Supplementary file 3 — Additional file 3. Cost-effectiveness model structure. Figure A: Health states and transitions within usual care. Figure B: Health states and transitions within the EDDIE intervention. [file 12877_2020_1904_MOESM3_ESM.pdf]
