# Supplementary material for: The effectiveness and cost effectiveness of a hospital avoidance program in a residential aged care facility: a prospective cohort study and modelled decision analysis
Source: BMC Geriatr. 2020 Dec 7;20:527. doi: 10.1186/s12877-020-01904-1 (PMC7720399; doi:10.1186/s12877-020-01904-1)

A: Density of the fitted Normal distribution for the annual number of hospital admissions: usual care period (orange dashed line) and intervention period (blue solid line)

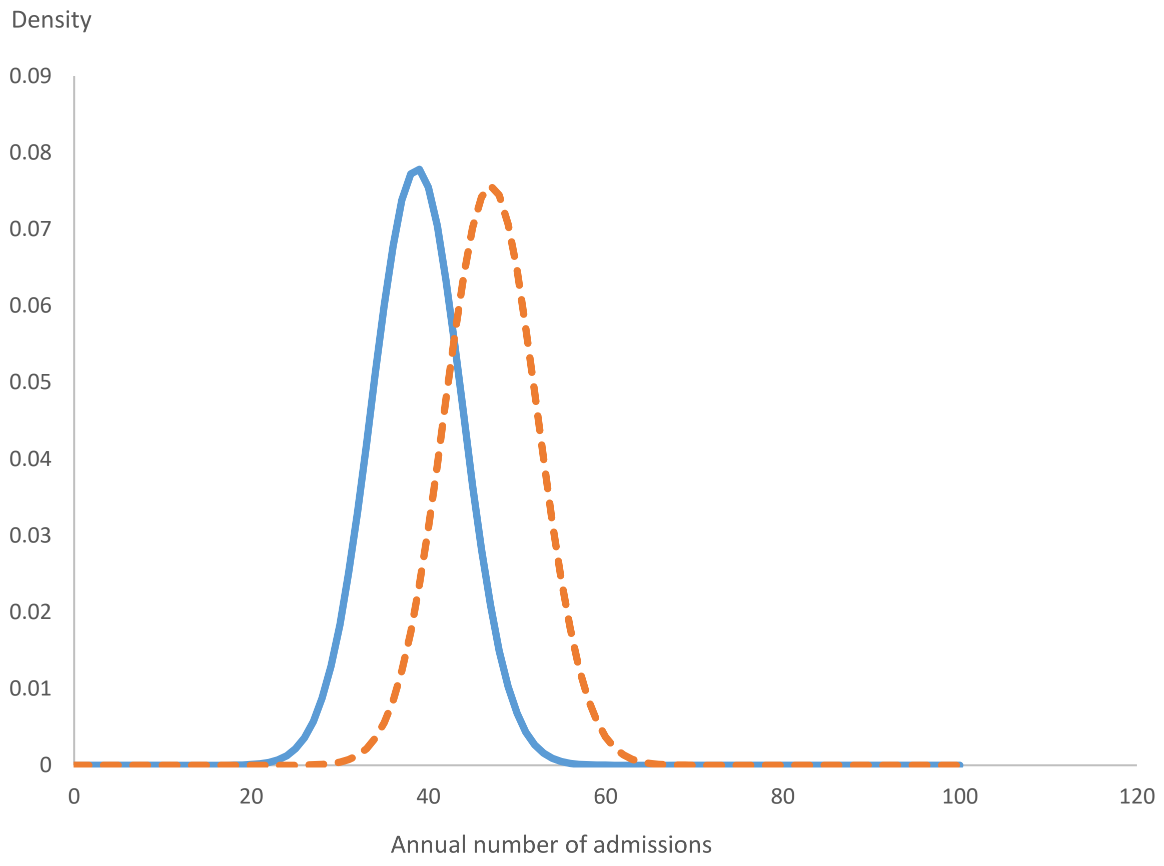

B: Density of the fitted Gamma distribution for length of stay: usual care cohort (orange dashed line) and intervention cohort (blue solid line).

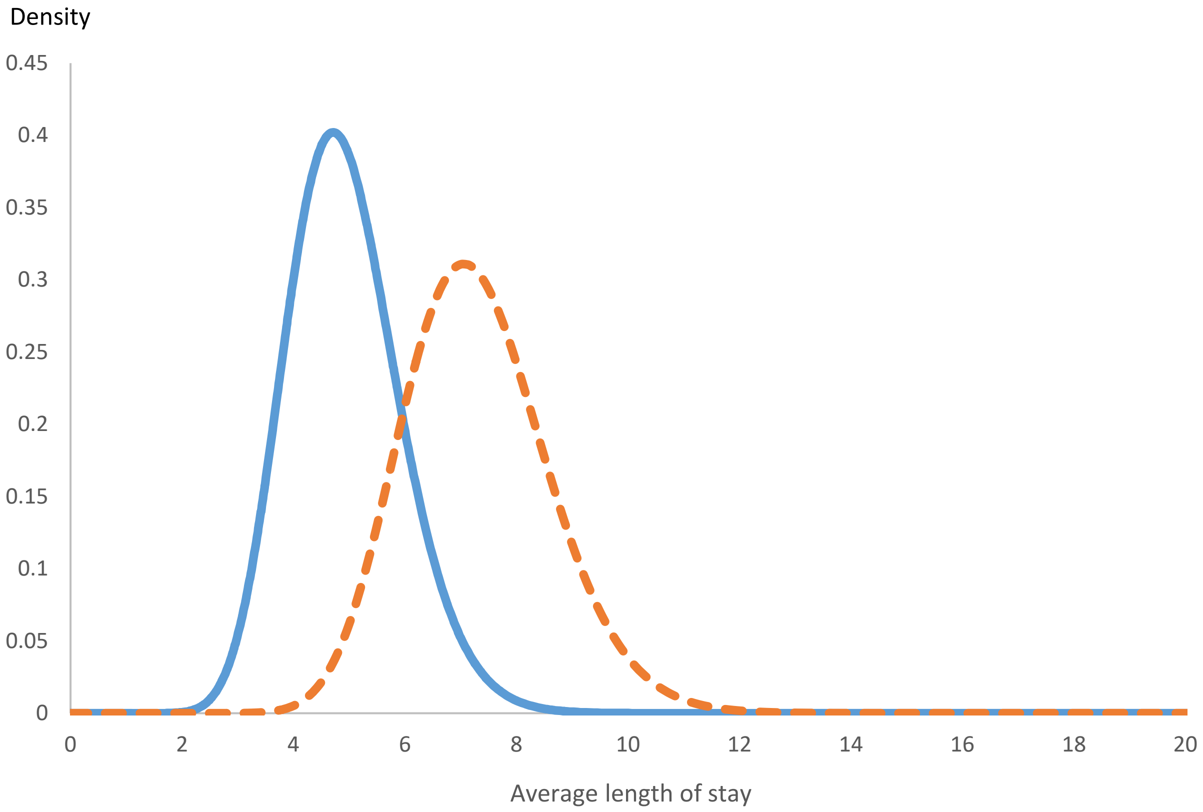

Supplement: Supplementary file 4 — Additional file 4. Figure A: Density of the fitted Normal distribution for the annual number of hospital admissions. Figure B: Density of the fitted Gamma distribution for length of stay. [file 12877_2020_1904_MOESM4_ESM.pdf]
